# Sex differences in coronary plaque changes assessed by serial computed tomography angiography

**DOI:** 10.1007/s10554-021-02204-4

**Published:** 2021-03-10

**Authors:** Mohammed El Mahdiui, Jeff M. Smit, Alexander R. van Rosendael, Danilo Neglia, Juhani Knuuti, Antti Saraste, Ronny R. Buechel, Anna Teresinska, Maria N. Pizzi, Albert Roque, Massimo Magnacca, Bart J. Mertens, Chiara Caselli, Silvia Rocchiccioli, Oberdan Parodi, Gualtiero Pelosi, Arthur J. Scholte

**Affiliations:** 1 Department of Cardiology, Leiden University Medical Center, Albinusdreef 2, 2300 RC Leiden, The Netherlands; 2 Fondazione Toscana Gabriele Monasterio, Viale Giuseppe Moruzzi 1 56124, Pisa, Italy; 3 Heart Center and PET Centre, Turku University Hospital and University of Turku, Turku, Finland; 4 Department of Nuclear Medicine, Cardiac Imaging, University Hospital and University of Zurich, Zurich, Switzerland; 5 National Institute of Cardiology, Warsaw, Poland; 6 Department of Cardiology, Hospital Universitari Vall D’Hebron, Barcelona, Spain; 7 Department of Radiology, Hospital Universitari Vall D’Hebron, Barcelona, Spain; 8 ASL12 U.O.C. Cardiologia, Viareggio, Italy; 9 Department of Medical Statistics, Leiden University Medical Center, Leiden, The Netherlands; 10 Institute of Clinical Physiology CNR, Viale Giuseppe Moruzzi 1 56124, Pisa, Italy; 11 Institute of Information Science and Technologies CNR, Pisa, Italy

**Keywords:** Coronary artery disease, Coronary computed tomography angiography, Sex, Menopause

## Abstract

Long-term data on sex-differences in coronary plaque changes over time is lacking in a low-to-intermediate risk population of stable coronary artery disease (CAD). The aim of this study was to evaluate the role of sex on long-term plaque progression and evolution of plaque composition. Furthermore, the influence of menopause on plaque progression and composition was also evaluated. Patients that underwent a coronary computed tomography angiography (CTA) were prospectively included to undergo a follow-up coronary CTA. Total and compositional plaque volumes were normalized using the vessel volume to calculate a percentage atheroma volume (PAV). To investigate the influence of menopause on plaque progression, patients were divided into two groups, under and over 55 years of age. In total, 211 patients were included in this analysis, 146 (69%) men. The mean interscan period between baseline and follow-up coronary CTA was 6.2 ± 1.4 years. Women were older, had higher HDL levels and presented more often with atypical chest pain. Men had 434 plaque sites and women 156. On a per-lesion analysis, women had less fibro-fatty PAV compared to men (β -1.3 ± 0.4%; p < 0.001), with no other significant differences. When stratifying patients by 55 years age threshold, fibro-fatty PAV remained higher in men in both age groups (p < 0.05) whilst women younger than 55 years demonstrated more regression of fibrous (β -0.8 ± 0.3% per year; p = 0.002) and non-calcified PAV (β -0.7 ± 0.3% per year; p = 0.027). In a low-to-intermediate risk population of stable CAD patients, no significant sex differences in total PAV increase over time were observed. Fibro-fatty PAV was lower in women at any age and women under 55 years demonstrated significantly greater reduction in fibrous and non-calcified PAV over time compared to age-matched men. (ClinicalTrials.gov number, NCT04448691.)

**Supplementary Information:**

The online version contains supplementary material available at 10.1007/s10554-021-02204-4.

## Introduction

Several studies have highlighted distinct sex-related differences for coronary artery disease (CAD). Women tend to be older when presenting with CAD [1], have lower rates of obstructive disease [2] but higher risk of major adverse cardiac events compared to men [2–5]. This discrepancy might arise from differences in plaque characteristics between men and women [6]. Postmortem histology studies reported plaque morphological differences between men and women [7–10]. However, in vivo intravascular studies have shown conflicting data regarding plaque burden and morphology between men and women [11–22]. These invasive studies were though performed in patients with an acute coronary syndrome (ACS), did not evaluate the plaques in the whole coronary tree or did not prospectively investigate sex differences in the natural plaque evolution over a long follow-up period. Coronary computed tomography angiography (CTA) allows for a fast and non-invasive assessment of coronary plaque burden and characterization of plaque composition comparable with intravascular ultrasound virtual histology (IVUS-VH) [23]. The aim of the current study was to evaluate the influence of sex on long-term in vivo plaque progression and evolution of plaque composition in a low-to-intermediate risk population in stable clinical conditions. Furthermore, the role of menopause on plaque progression and composition was also evaluated.

## Materials and methods

### Study design

The SMARTool (Simulation Modeling of coronary ARTery disease: a tool for clinical decision support, Horizon 2020) project, is a prospective, international, multicenter study with the aim of integrating clinical, molecular, cellular and imaging data to provide a patient-specific risk stratification model exploitable for clinical decision support in stable CAD management [24, 25]. Patients who had undergone a coronary CTA at baseline for suspected CAD were prospectively included and subsequently underwent a follow-up coronary CTA. Patients with stable CAD without a history of myocardial infarction, heart failure or surgical procedures related to heart diseases were included. The complete inclusion and exclusion criteria are provided in the supplementary material.

### Study population

**Fig. 1:**
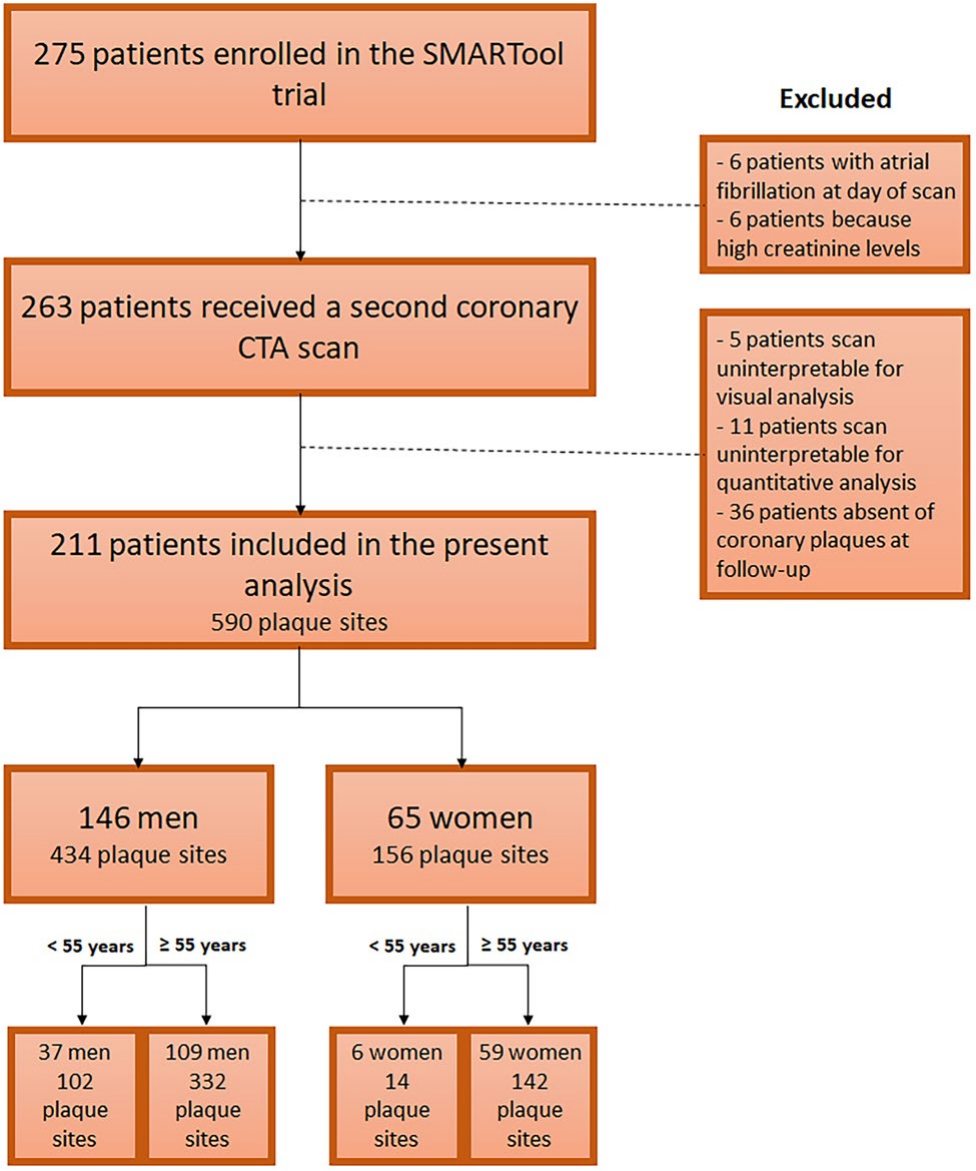
Flow diagram of the study population

Patients who had undergone clinically indicated coronary CTA in the period 2009–2012 or were part of the EVINCI (FP7-222,915) or the ARTreat (FP7-224,297) clinical studies were included. The Diamond-Forrester model was used to estimate the pretest probability of CAD [26]. Inclusion and exclusion criteria have been previously [25]. Data on cardiovascular risk factors and medical therapy were prospectively collected at baseline and follow-up. Statin intensity was classified according to the American College of Cardiology and American Heart Association guidelines [27]. In total, 275 patients from 5 European countries (Finland, Italy, Poland, Spain and Switzerland) were recruited in 7 centers. Of the 263 patients who underwent a follow-up coronary CTA, 52 patients were excluded because of uninterpretable coronary CTA for visual (n = 5) or quantitative CTA analysis (n = 11) or absence of coronary plaques at follow-up (n = 36). Thus, 211 patients were finally included in the present analysis (Fig. 1).

### Coronary CTA analysis protocol

The coronary CTA protocol has been described previously [25]. In brief, anonymized coronary CTA data were transferred to a core laboratory (Leiden University Medical Center) for visual and quantitative analysis (supplementary material) and researchers were blinded to patients clinical data. Quantitative analysis was performed on visually identified plaques using a dedicated software package (QAngio CT Research Edition version 3.1.2.0, Medis Medical Imaging Systems, Leiden, the Netherlands). The software automatically detects the centerline, lumen and the vessel wall and allows the user for manual adjustment if needed [23, 28]. The baseline and follow-up coronary CTA were analyzed side-by-side and lesions were identified using anatomical markers. Several parameters were derived from the quantitative analysis: percentage diameters stenosis, lesion length, remodeling index, total vessel volume, total plaque volume and plaque composition volumes. Plaque composition volumes were determined using predefined Hounsfield units (HU) cutoff values: > 350 HU for calcified plaque and -30 to 350 HU for non-calcified plaque. Non-calcified plaque was further classified in necrotic core plaque (-30 to 75 HU), fibro-fatty plaque (76 to 130 HU) and fibrous plaque (131 to 350 HU). Total plaque volume and plaque composition volumes were normalized for the vessel volume and the percentage atheroma volume (PAV) calculated as follows: (plaque volume/ total vessel volume) × 100% and reported as a percentage. The inter- and intra-observer variability have been described previously [28–30].

### Statistical analysis

Continuous variables are expressed as mean ± standard deviation (SD) if normally distributed and median and interquartile range (IQR) if non-normally distributed. Normality was assessed using histograms and Q-Q plots. Categorical variables are presented as frequencies and percentages and compared using the Chi square test or the Fisher’s exact test. Normally distributed continuous variables were compared using the Student’s *t*-test and the Mann–Whitney *U*-test if not normally distributed. Quantitative analysis parameters were compared on a per-lesion basis. Analysis of annual rate of lesion progression was performed using linear mixed models (LMM) to correct for per lesion and per patient factors. Fixed effects in the models included sex, interscan period and the interaction between sex and interscan period. In addition, the LMM was adjusted for age, hypertension, diabetes mellitus, smoking, family history of CAD, obesity, LDL and HDL at baseline. Random effects included intercept and an unstructured covariance was used to account for within-patient and within-plaque correlation over time. A sub-analysis was performed in patients aged under and over 55 years at baseline coronary CTA scan to assess the influence of menopause on plaque progression in women compared to men. The models provide a test for systematic between-group difference across time, as well as a test for between-group differences in the trend. The estimated difference (β) of women compared to men and the interaction are presented with standard error (SE), 95% confidence interval (CI) and p-values. Statistical analyses were performed using SPSS version 25.0 (SPSS, Armonk, NY) and a two-sided p-value < 0.05 was considered statistically significant.

## Results

### Baseline patient characteristics

Of the 211 patients included in the present analysis, 146 (69%) were men and 65 (31%) were women. Women were generally older, had higher HDL levels and presented more often with atypical chest pain. The mean interscan period between baseline and follow-up coronary CTAs was 6.2 ± 1.4 years (minimum 1.9- maximum 11.3). Baseline patient characteristics are shown in Table 1. When stratifying the population according to age groups, 43 (20%) were under 55 years at the time of baseline coronary CTA scan and 168 (80%) were 55 years or older.

**Table 1:** Patient characteristics

Variables	Total (n = 211)	Men (n = 146)	Women (n = 65)	p-value
**Clinical**				
Age, years	62 ± 8	61 ± 8	64 ± 7	**0.001**
Body mass index, kg/m^2^	27.6 ± 3.8	27.6 ± 3.4	27.5 ± 4.5	0.835
Family history of CAD	96 (46)	59 (40)	37 (57)	**0.049**
Current smoker	33 (16)	25 (17)	8 (12)	0.306
Diabetes mellitus	41 (19)	25 (17)	16 (25)	0.266
Dyslipidemia	138 (65)	91 (62)	47 (72)	0.305
Hypertension	136 (65)	90 (62)	46 (71)	0.370
Chest pain				
Typical	47 (22)	34 (23)	13 (20)	0.310
Atypical	96 (46)	56 (38)	40 (62)	**0.017**
Non-anginal	1 (1)	1 (1)	0 (0)	1.000
**Medication**				
ACE-inhibitors/ARB’s	96 (46)	64 (44)	32 (49)	0.839
Aspirin	133 (63)	90 (62)	43 (66)	0.891
Beta-blockers	86 (41)	55 (38)	31 (48)	0.366
Diuretics	32 (15)	13 (9)	19 (29)	** < 0.001**
Statin therapy				
Statins at baseline	112 (53)	74 (51)	38 (59)	0.296
High-intensity	7 (6)	4 (5)	3 (8)	0.687
Low-/Moderate-intensity	34 (30)	25 (34)	9 (24)	0.271
Statins at follow-up	145 (69)	105 (72)	40 (62)	0.133
High-intensity	27 (19)	19 (19)	8 (20)	0.792
Low-/Moderate-intensity	110 (76)	78 (77)	32 (80)	0.472
**Biochemical**				
Creatinine, mg/dl	0.873 ± 0.197	0.943 ± 0.174	0.734 ± 0.166	** < 0.001**
Glucose, mg/dl	109.51 ± 26.63	110.55 ± 26.80	107.42 ± 26.38	0.458
Triglycerides, mg/dL	121.93 ± 62.51	126.92 ± 65.04	111.66 ± 56.13	0.125
Total Cholesterol, mg/dL	185.52 ± 48.32	182.55 ± 48.29	192.23 ± 48.10	0.190
LDL, mg/dL	110.28 ± 41.24	108.35 ± 41.42	114.65 ± 40.84	0.318
HDL, mg/dL	51.33 ± 14.87	49.28 ± 14.53	55.97 ± 14.69	**0.003**

Bold indicates statistical signifcance of p value < 0.05

Patient characteristics are at baseline unless otherwise indicated. Values are presented as mean ± standard deviation or n (%)

*ACE* angiotensin-converting enzyme, *ARB* angiotensin-II-receptor blocker, *CAD* coronary artery disease, *HDL* high-density lipoprotein, *LDL* low-density lipoprotein

### Baseline plaque characteristics and changes of total and compositional PAV

**Fig. 2:**
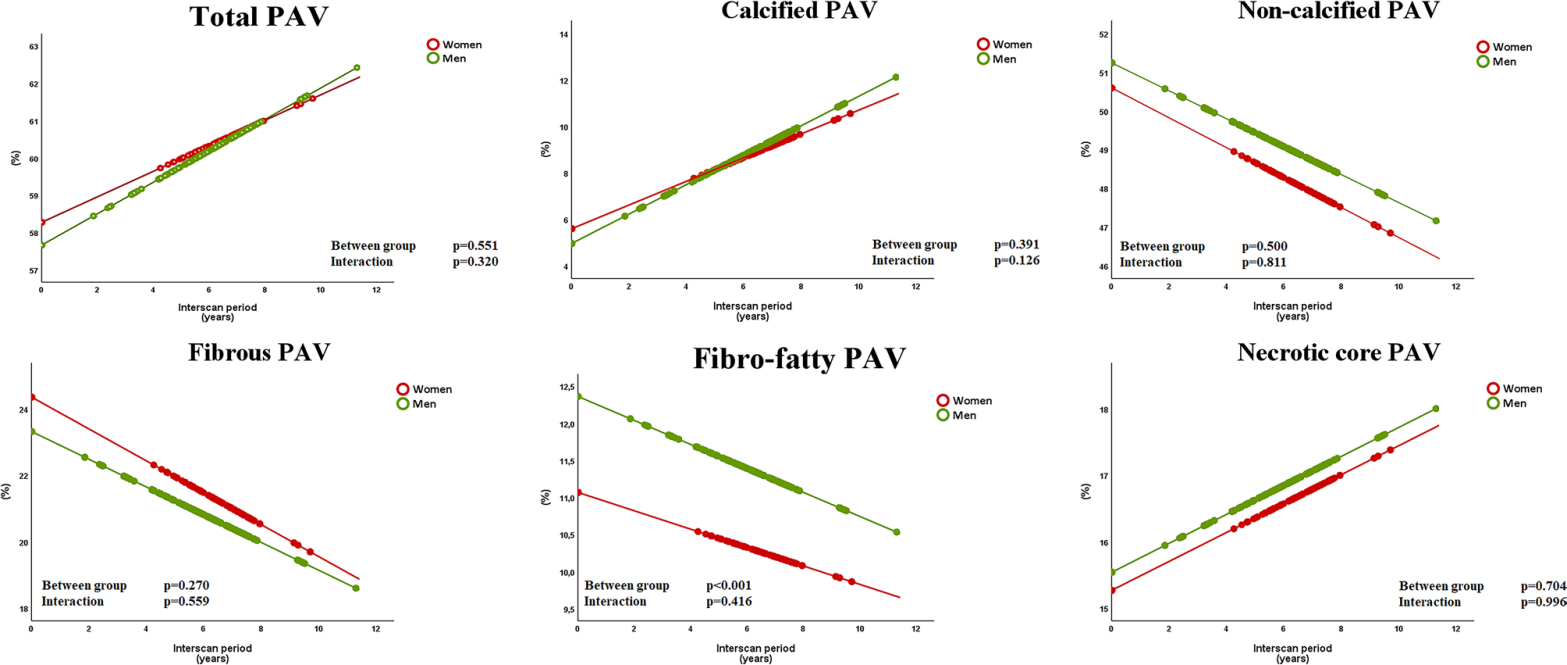
Plaque changes on a per-lesion analysis shown for women and men. The line graphs represent the estimated average trend from baseline to 12 years for both groups based on a linear mixed modelling, with tests for the systematic between-group differences as well as for differences in trend. Circles represent the estimated mean percentage at the time point the follow-up scan was performed. *PAV* percentage atheroma volume

**Fig. 3:**
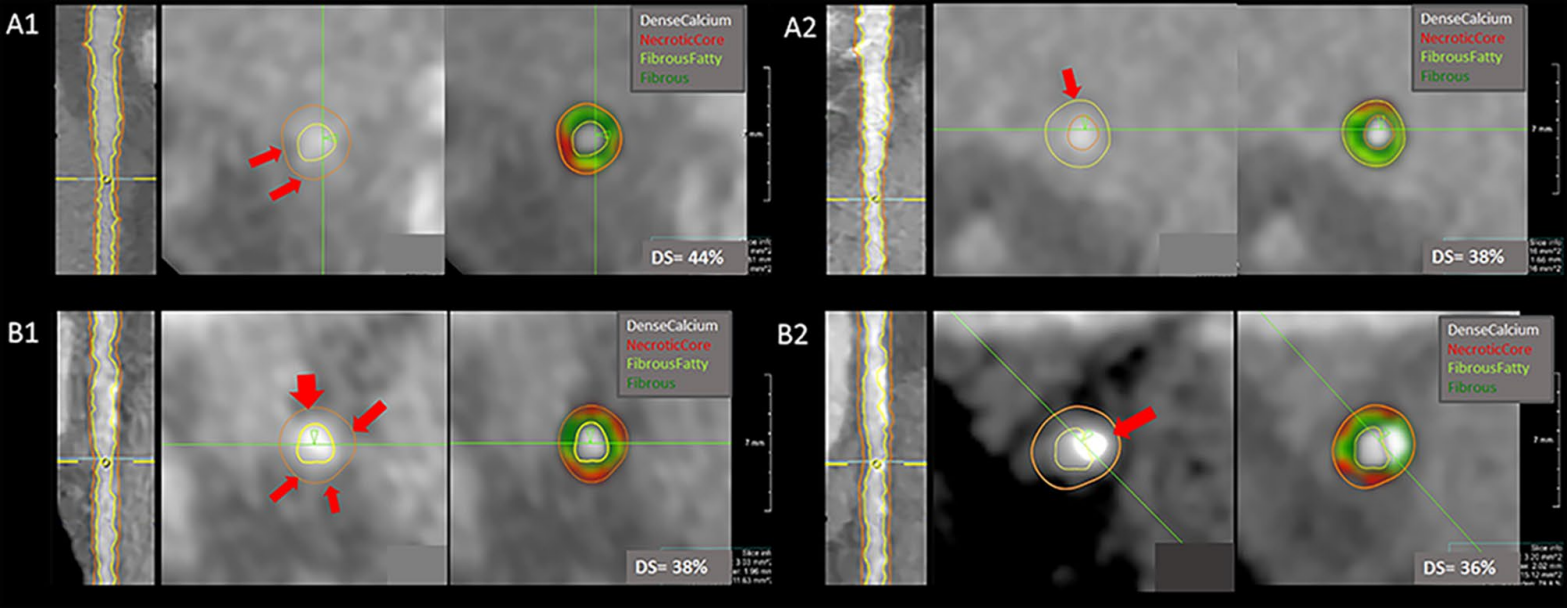
Quantitative assessment of coronary plaques in a male and female patient at baseline and follow-up. Panel A represents quantitative coronary plaque analysis of a 62-year-old male patient of the mid-left anterior descending artery at baseline (A1) and after 5.4 years follow-up (A2). During follow-up reduction of necrotic core and an increase in fibrous and fibrous fatty can be observed. Panel B represents quantitative coronary plaque analysis of a 58-year-old female patient of the proximal circumflex artery at baseline (B1) and after 5.9 years follow-up (B2). A reduction of necrotic core and the formation of dense calcium can be observed during follow-up. *DS* diameter stenosis

A total of 590 plaques were identified, 434 (74%) plaques were found in men and 156 (26%) in women. Baseline plaque characteristics are shown in Table 2. At baseline men had higher degree of stenosis (p < 0.05). Men also had higher absolute volumes of fibro-fatty and necrotic core (p < 0.05), but after correction for vessel volume only fibro-fatty PAV remained higher in men (p < 0.001). Table 3 summarizes the differences in plaque changes between men and women. Total PAV increased 0.42%/ per lesion/ per year and 0.34%/ per lesion/ per year, in men and women respectively, no difference in the progression was observed (β -0.1 ± 0.1 (95% CI -0.2 to 0.1) % per year; p = 0.320). Similarly, no sex differences in compositional changes were observed, although women had less fibro-fatty PAV per-lesion compared to men during follow-up (β -1.3 ± 0.4 (95% CI -2.0 to -0.6) %; p < 0.001), despite no difference in the rate of plaque progression compared to men (p = 0.416) (Fig. 2). Examples of quantitative coronary plaque analysis are demonstrated in Fig. 3.

**Table 2:** Plaque characteristics at baseline

Variables	Total (n = 590)	Men (n = 434)	Women (n = 156)	p-value
Lesion length, mm	13.3 (6.5–30.5)	13.4 (6.6–31.6)	13.1 (6.1–24.9)	0.196
Diameter stenosis, %	23.8 (14.5–32.8)	24.6 (14.9–33.5)	21.5 (13.3–30.8)	**0.044**
Remodeling index	0.85 ± 0.16	0.85 ± 0.16	0.85 ± 0.15	0.973
Total vessel volume, mm^3^	247.7 (116.2–528.1)	252.1 (123.5–550.0)	229.6 (101.3–426.4)	0.072
Total plaque volume, mm^3^	141.0 (67.5–302.8)	143.3 (70.6–322.3)	133.2 (60.0–239.4)	0.094
Calcified plaque volume, mm^3^	7.7 (1.7–23.0)	7.3 (1.7–22.4)	8.5 (1.8–23.3)	0.659
Non-calcified plaque volume, mm^3^	123.3 (57.3–269.7)	128.0 (58.7–284.1)	114.7 (52.4–205.7)	0.082
Fibrous plaque volume, mm^3^	53.6 (24.7–113.4)	54.3 (24.7–119.1)	50.9 (23.8–105.6)	0.383
Fibro-fatty plaque volume, mm^3^	27.8 (12.9–63.1)	29.3 (13.2–68.9)	24.5 (11.0–49.8)	**0.009**
Necrotic core plaque volume, mm^3^	34.4 (15.1–75.4)	37.3 (16.0–82.3)	30.2 (12.5–62.8)	**0.032**
Total PAV, %	57.9 ± 7.8	57.7 ± 7.7	58.3 ± 7.9	0.453
Calcified PAV, %	3.4 (0.8–7.7)	3.2 (0.8–7.5)	4.1 (1.4–8.3)	0.062
Non-calcified PAV, %	50.6 ± 8.6	50.8 ± 9.0	50.0 ± 7.4	0.304
Fibrous PAV, %	23.0 ± 8.1	22.7 ± 8.1	23.8 ± 8.2	0.167
Fibro-fatty PAV, %	12.0 ± 3.4	12.4 ± 3.5	10.9 ± 2.8	** < 0.001**
Necrotic core PAV, %	15.6 ± 6.6	15.7 ± 6.5	15.3 ± 6.9	0.492

Bold indicates statistical signifcance of p value < 0.05

Values are presented as mean ± standard deviation or median (interquartile range)

*PAV* percentage atheroma volume

**Table 3:** Plaque morphological and compositional changes on a per-lesion analysis shown for women compared to men

Variables	Total (n = 590) β ± SE (95% CI)	p-value
Lesion length, mm		
Between group comparison	− 4.4 ± 2.8 (− 9.9 to 1.1)	0.116
Interaction	− 0.0 ± 0.0 (− 0.0 to 0.0)	0.744
Diameter stenosis, %		
Between group comparison	− 0.0 ± 0.0 (− 0.1 to 0.0)	0.061
Interaction	0.0 ± 0.0 (− 0.0 to 0.0)	0.981
Remodeling Index		
Between group comparison	0.0 ± 0.0 (− 0.1 to 0.0)	0.758
Interaction	− 0.0 ± 0.0 (− 0.0 to 0.0)	0.121
Total PAV, %		
Between group comparison	0.6 ± 1.0 (− 1.4 to 2.6)	0.551
Interaction	− 0.1 ± 0.1 (− 0.2 to 0.1)	0.320
Calcified PAV, %		
Between group comparison	0.6 ± 0.7 (− 0.8 to 2.1)	0.391
Interaction	− 0.1 ± 0.1 (− 0.3 to 0.0)	0.126
Non-calcified PAV, %		
Between group comparison	− 0.6 ± 1.0 (− 2.5 to 1.2)	0.500
Interaction	− 0.0 ± 0.1 (− 0.2 to 0.2)	0.811
Fibrous PAV, %		
Between group comparison	1.0 ± 0.9 (− 0.8 to 2.9)	0.270
Interaction	− 0.1 ± 0.1 (− 0.3 to 0.1)	0.559
Fibro-fatty PAV, %		
Between group comparison	− 1.3 ± 0.4 (− 2.0 to − 0.6)	** < 0.001**
Interaction	0.0 ± 0.0 (− 0.1 to 0.1)	0.416
Necrotic core PAV, %		
Between group comparison	− 0.3 ± 0.7 (− 1.7 to 1.1)	0.704
Interaction	− 0.0 ± 0.1 (− 0.2 to 0.2)	0.996

Bold indicates statistical signifcance of p value < 0.05

Values are presented as estimates (β) ± standard error (SE) (95% confidence interval)

*CI* confidence interval, *PAV* percentage atheroma volume

### Sex differences and the role of menopause on plaque progression

**Fig. 4:**
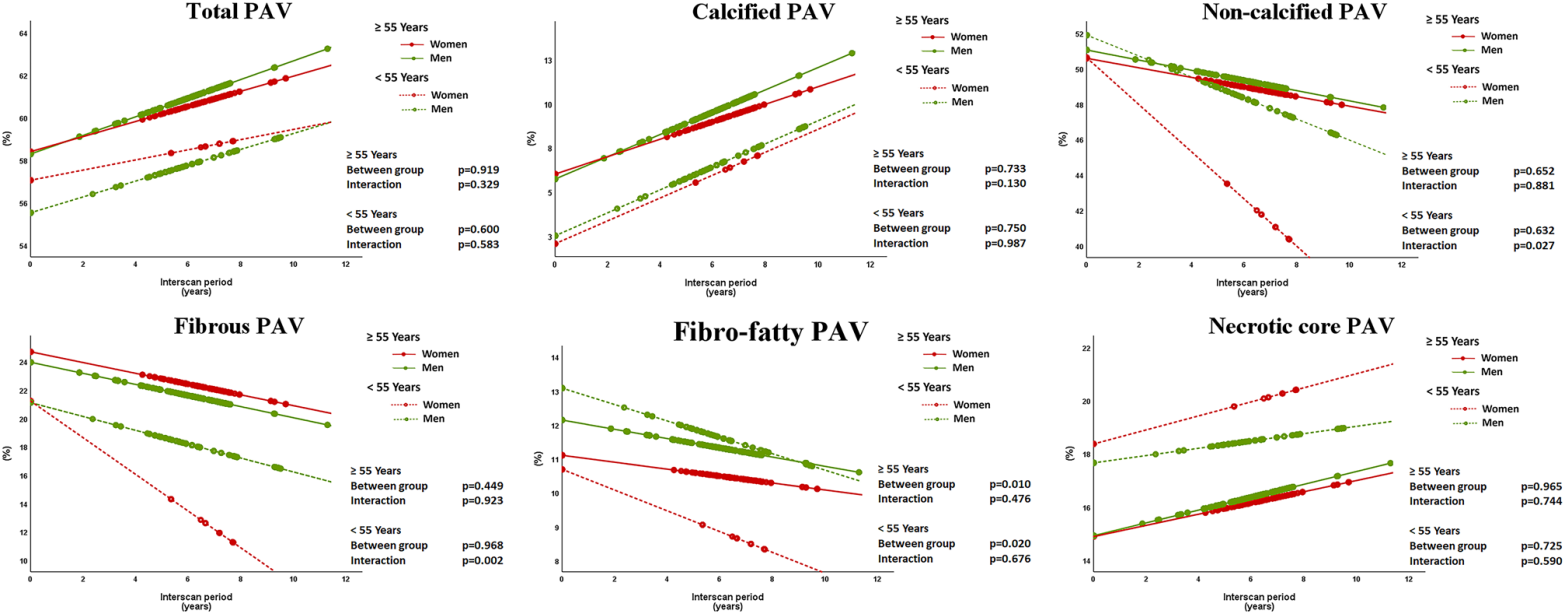
Plaque changes on a per-lesion analysis shown for women and men stratified according to the age group (< 55 vs ≥ 55 years old). The line graphs represent the estimated average trend from baseline to 12 years for both groups based on a linear mixed modelling, with tests for the systematic between-group differences as well as for differences in trend. Circles represent the estimated mean percentage at the time point the follow-up scan was performed. *PAV* percentage atheroma volume

Table 4 summarizes the differences in plaque progression between men and women stratified according to age (< 55 vs ≥ 55 years). Women had less fibro-fatty PAV in both age groups (< 55 vs ≥ 55 years) compared to men (p < 0.05). Women younger than 55 years showed more regression of fibrous PAV (β -0.8 ± 0.3 (95% CI -1.3 to -0.3) % per year; p = 0.002) and non-calcified PAV (β -0.7 ± 0.3 (95% CI -1.4 to -0.1) % per year; p = 0.027), compared to men. These differences were absent in the age group ≥ 55 years old (Fig. 4).

**Table 4:** Plaque morphological and compositional changes on a per-lesion analysis shown for women compared to men stratified according to < 55 or ≥ 55 years of age

Variables	< 55 years (n = 112) β ± SE (95% CI)	p-value	≥ 55 years (n = 478) β ± SE (95% CI)	p-value
Lesion length, mm				
Between group comparison	− 9.0 ± 9.2 (− 27.5 to 9.4)	0.329	− 4.2 ± 2.9 (− 10.1 to 1.6)	0.151
Interaction	0.0 ± 0.1 (− 0.1 to 0.1)	0.871	− 0.0 ± 0.0 (− 0.1 to 0.0)	0.580
Diameter stenosis, %				
Between group comparison	− 0.0 ± 0.0 (− 0.1 to 0.1)	0.659	− 0.0 ± 0.0 (− 0.1 to 0.0)	**0.031**
Interaction	0.0 ± 0.0 (− 0.0 to 0.0)	0.965	0.0 ± 0.0 (− 0.0 to 0.0)	0.823
Positive remodeling				
Between group comparison	− 0.0 ± 0.1 (− 0.1 to 0.1)	0.649	0.0 ± 0.0 (− 0.0 to 0.0)	0.563
Interaction	− 0.0 ± 0.0 (− 0.0 to 0.0)	0.487	− 0.0 ± 0.0 (− 0.0 to 0.0)	0.125
Total PAV, %				
Between group comparison	1.5 ± 2.9 (− 4.3 to 7.3)	0.600	0.1 ± 1.1 (− 2.1 to 2.3)	0.919
Interaction	− 0.1 ± 0.2 (− 0.6 to 0.3)	0.583	− 0.1 ± 0.1 (− 0.2 to 0.1)	0.329
Calcified PAV, %				
Between group comparison	− 0.5 ± 1.4 (− 3.3 to 2.4)	0.750	0.3 ± 0.8 (− 1.3 to 1.9)	0.733
Interaction	− 0.0 ± 0.2 (− 0.4 to 0.4)	0.987	− 0.1 ± 0.1 (− 0.3 to 0.0)	0.130
Non-calcified PAV, %				
Between group comparison	− 1.3 ± 2.6 (− 6.6 to 4.0)	0.632	− 0.5 ± 1.0 (− 2.5 to 1.6)	0.652
Interaction	− 0.7 ± 0.3 (− 1.4 to − 0.1)	0.027	0.0 ± 0.1 (− 0.2 to 0.2)	0.881
Fibrous PAV, %				
Between group comparison	0.1 ± 2.8 (− 5.4 to 5.7)	0.968	0.7 ± 1.0 (− 1.2 to 2.7)	0.449
Interaction	− 0.8 ± 0.3 (− 1.3 to − 0.3)	0.002	0.0 ± 0.1 (− 0.2 to 0.2)	0.923
Fibro-fatty PAV, %				
Between group comparison	− 2.4 ± 1.0 (− 4.4 to − 0.4)	0.020	− 1.0 ± 0.4 (− 1.8 to − 0.3)	**0.010**
Interaction	− 0.1 ± 0.2 (− 0.4 to 0.2)	0.676	0.0 ± 0.0 (− 0.1 to 0.1)	0.476
Necrotic core PAV, %				
Between group comparison	0.7 ± 2.0 (− 3.3 to 4.8)	0.725	− 0.0 ± 0.8 (− 1.5 to 1.4)	0.965
Interaction	0.1 ± 0.2 (− 0.3 to 0.6)	0.590	− 0.0 ± 0.1 (-0.2 to 0.2)	0.744

Bold indicates statistical signifcance of p value < 0.05

Values are presented as estimates (β) ± standard error (SE) (95% confidence interval)

*CI* confidence interval, *PAV* percentage atheroma volume

## Discussion

In this prospective and multicenter study of serial coronary CTA we demonstrated that fibro-fatty PAV was higher in men compared to women at any age. During long-term follow-up no sex differences were detected in the change of total or compositional PAV on a per-lesion analysis after correction for multiple cardiovascular risk factors. However, when stratifying patients according to age groups (< 55 vs ≥ 55 years), coronary plaques in women younger than 55 years demonstrated more pronounced reduction of fibrous and non-calcified PAV compared to age-matched men. These results provide further insight in the understanding of the role of sex on long-term evolution of plaque morphology in stable CAD.

Similar to previous studies, we found that women had fewer lesions compared to men [8, 15]. However, the total PAV per-lesion at baseline was comparable for men and women, which was also demonstrated in several other studies using IVUS [11, 14, 15, 31]. In a sub analysis of the PROSPECT (Providing Regional Observations to Study Predictors of Events in the Coronary Tree) trial, women had fewer lesions and fewer diseased vessels than men, yet comparable plaque burden on a per-lesion analysis. More importantly, we did not find sex differences in the progression of total PAV during long-term follow-up. Few studies have investigated the influence of sex on quantitatively assessed plaque progression. In a population of 727 men and 251 women, Nicholls et al. also demonstrated no sex differences in the progression of total PAV using IVUS during a follow-up of 18–24 months [12]. Plaque compositional differences between men and women were first reported from limited postmortem studies in patients with advanced CAD and demonstrated coronary plaques in women, especially young women, contained less dense fibrous tissue compared to men [7, 8]. More recently, IVUS-VH studies in patients with ACS demonstrated women tend to have lower fibrous tissue compared to men [14, 15]. This is in agreement with our findings of a greater reduction of fibrous PAV in women younger than 55 years compared to age-matched men. Absolute values of fibro-fatty PAV were higher in men compared to women in both age groups, and at both CTA scan time points, as previously described[15] but its change in time did not differ between men and women. Non-calcified PAV regressed more in women younger than 55 years than in younger men, without any difference in subjects of 55 years or older. Given the known association between non-calcified plaques with ischemia and ACS, these findings might partially explain the lower risk of symptomatic CAD in young women [32–34].

Cardiovascular diseases are increased in women after menopause and the loss of protective female sex hormones has been suggested to play an important role [35]. Sex hormones demonstrate a wide range of effects on endothelial cells, vascular tone, lipids, coagulation and cardiomyocytes [35]. Consequently, several large randomized trials were conducted to investigate hormone replacement therapy (HRT) following menopause for reducing risk of cardiovascular disease. Although the Women’s Health Initiative trials demonstrated no benefit of HRT initiated late after menopause on cardiovascular events [36, 37], other trials demonstrated that timely starting of HRT was associated with lower progression of atherosclerosis, but did not find evidence for an effect on coronary atherosclerosis progression [38–41]. Our findings of a similar progression of total PAV between men and women in both age groups, but differences in compositional changes between men and women younger than 55 years but not in those of 55 years or older is a new insight. Although previous trials have not demonstrate an effect of HRT on total coronary atherosclerosis changes, our findings suggest coronary plaques should be evaluated for compositional changes following HRT. HRT might potentially positively influence plaque compositional changes.

### Clinical implications

The higher regression of fibrous and non-calcified PAV in women compared to men younger than 55 years old is an clinically important finding. Non-calcified plaques are associated with ischemia and ACS. [28–30] The absence of this difference in the, likely post-menopausal, women of 55 years or older hints to a slowing of the regression of non-calcified PAV to match that of the men and thereby increasing the risk for symptomatic CAD. Several strategies could be considered for this increased risk. Monitoring and treatment of cardiovascular risk factors of women around the age of menopause could be employed. Coronary CTA with quantitative plaque assessment might provide additional information on risk for future symptomatic CAD which could prompt early treatment of cardiovascular risk factors. Moreover, HRT might potentially positively influence plaque compositional changes and should be investigated.

### Study limitations

Similar to other trials, women were underrepresented in our study. We used 55 years as a proxy for menopause, since menopause status was unavailable from clinical records. Although, the mean age of menopause has been demonstrated to be lower than 55 years, we cannot exclude the fact that premenopausal subjects might have been included in the ≥ 55 years age group [42]. Furthermore, information on HRT or sex hormone levels, which might have added relevant information, was also unavailable. A relative limited number of patients were included in this study and the sub analysis of sex differences in the different age groups should be interpreted with caution. As coronary CTA scanners from different vendors were used, a predefined standard operating procedure was applied to minimize variances among centers and quantitative analysis was performed in the core lab exclusively on visually recognized plaques: however, although careful visual examination was performed in the whole coronary tree, some plaques might have been unrecognized.

## Conclusions

In a low-to-intermediate risk population of stable CAD with serial CTA scan during a follow-up of 6.2 ± 1.4 years women younger than 55 years demonstrated, after correction for several cardiovascular risk factors, a more pronounced reduction of fibrous and non-calcified PAV compared to age-matched men. No differences in the change of total or compositional PAV were observed between women and men of 55 years or older. Finally, the absolute value of fibro-fatty PAV was consistently higher in men than in women at any age.

## Supplementary Information

Below is the link to the electronic supplementary material.

Supplementary file1 (DOCX 121 KB)

## Abbreviations

ACS: acute coronary syndrome
CAD: coronary artery disease
CTA: computed tomography angiography
HRT: hormone replacement therapy
HU: Hounsfield units
IVUS-VH: intravascular ultrasound virtual histology
PAV: percentage atheroma volume
