# Supplementary material for: Sex differences in coronary plaque changes assessed by serial computed tomography angiography
Source: Int J Cardiovasc Imaging. 2021 Mar 10;37(7):2311–21. doi: 10.1007/s10554-021-02204-4 (PMC8286938; doi:10.1007/s10554-021-02204-4)
Supplement: Supplementary file 1 — Supplementary file1 (DOCX 121 KB) [file 10554_2021_2204_MOESM1_ESM.docx]

**SUPPLEMENTARY MATERIAL**

**Supplemental methods 1.**

**Standard operating procedure (SOP) for coronary CTA in SMARTool**

- The CT image quality in the majority of cases should be preferably 2.

- Absence of motion or other artifacts in the acquired image; heart rate less than 65 beats/min and optimally less than 60 beats/min.
- Please administer nitroglycerin prior to the CTA acquisition.
- Optimized reconstruction of the most suitable cardiac cycle (i.e. diastole at 70-80% of the R-R interval).
- Please send multiple cardiac phases so we may choose different phases for different coronary segments if needed.
- kV, mA and contrast protocol should be preferably the same for the first and second scan. However, changes in patient body composition and local acquisition protocols should guide decision making.
- The reconstructed field of view should be reduced to maximize number of pixels devoted to depiction of the heart, usually field of view of 200-250 mm for coronary CTA studies of native coronary arteries.

**Supplemental methods 2.**

**Full list of eligibility, inclusion, exclusion and exit criteria of SMARTool Clinical Study**

**Eligibility criteria:**

A. Clinical history and lifestyle data records available at one-time point.

B. At least one previous CCTA examination performed for suspected CHD and of good quality to allow for: a) Non-invasive FFR-CT assessment b) Quantitative (automated) 17 segments (AHA) analysis and measurement with ≤10% error of MLA (mm2), lumen area stenosis (%), mean plaque burden (mm3), plaque burden at MLA (%), and remodeling index, c) Plaque phenotype assessment: HU based classification in calcified, non-calcified (LAP) and mixed, napkin-ring sign, CAC score.

C. Previous blood and plasma sample available for retrospective analysis

**Inclusion criteria:**

1) male and female subjects

2) aged 45-82 years

3) Caucasian population

4) submitted to CCTA for suspected CHD between 2009 and 2012 (in the context of EVINCI and ARTreat FPVII studies) at the Hospitals reported in “SMARTool Clinical Center” document and satisfying the elegibility criteria reported above

5) submitted to clinical Follow-up in the last 6 months with stable clinical conditions and documented CHD or persistent intermediate/high probability of CHD

6) Signed informed consents (clinical and genetic)

**Exclusion criteria:**

1) Multi-vessel severe disease (3 vessels and/or LM disease with >90% stenosis).

2) Severe coronary calcification (CAC score > 600).

3) Having undergone surgical procedures related to heart diseases (valve replacement, CRT or CRTD treatment, any surgery of the heart or arteries).

4) Documented MACE at history (myocardial infarction, severe heart failure, recurrent angina) in the last 6 months with/without revascularization

5) Documented severe peripheral vascular disease (carotid, femoral)

6) Surgery of carotid and/or peripheral arteries or cerebral ischemic attack

7) History/surgery of Abdominal Aortic Aneurysm(AAA).

8) Severe Heart failure (NYHA Class III-IV)

9) LV dysfunction (left ventricle EF <40%).

10) Atrial fibrillation.

11) Lack of written informed consent (clinical consent and/or genetic consent)

12) Pregnancy (evaluated by urine test) and breastfeeding

13) Active Cancer

14) Asthma

15) Cardiomyopathy or congenital heart disease

16) Significant valvular disease (hemodynamically significant valvular stenosis or insufficiency by echoDoppler)

17) Renal dysfunction (creatinine > 1.3 mg/dL)

18) Chronic Kidney Disease (eGFR < 30 ml/min/1.73 m2)

19) Hepatic failure (at least 3 of the following: albumin < 3.5 g/dL; prolonged prothrombin time–PT; jaundice; ascites)

20) Waldenstrom disease

21) Multiple myeloma

22) Autoimmune/Acute inflammatory disease

23) Previous severe adverse reaction to iodine contrast agent

24) Positivity at blood tests for HIV, Hepatitis B and C (CRF number 1-clinical evaluation)

**Exit Criteria:**

A) Informed consent retired by the patient (genetic or clinical)

B) Adverse events to contrast medium during CCTA
